# Gut microbiome communication with bone marrow regulates susceptibility to amebiasis

**DOI:** 10.1172/JCI133605

**Published:** 2020-06-22

**Authors:** Stacey L. Burgess, Jhansi L. Leslie, Jashim Uddin, David N. Oakland, Carol Gilchrist, G. Brett Moreau, Koji Watanabe, Mahmoud Saleh, Morgan Simpson, Brandon A. Thompson, David T. Auble, Stephen D. Turner, Natasa Giallourou, Jonathan Swann, Zhen Pu, Jennie Z. Ma, Rashidul Haque, William A. Petri

**Affiliations:** 1 Division of Infectious Diseases and International Health, Department of Medicine, University of Virginia School of Medicine, Charlottesville, Virginia, USA.; 2 AIDS Clinical Center, National Center for Global Health and Medicine, Shinjuku, Tokyo, Japan.; 3 Department of Biochemistry and Molecular Genetics and; 4 Department of Public Health Sciences, University of Virginia School of Medicine, Charlottesville, Virginia, USA.; 5 Division of Integrative Systems Medicine and Digestive Diseases, Imperial College London, London, United Kingdom.; 6 Department of Statistics and; 7 Department of Public Health Sciences, University of Virginia, Charlottesville, Virginia, USA.; 8 International Centre for Diarrhoeal Diseases Research, Dhaka, Bangladesh.

**Keywords:** Immunology, Infectious disease, Hematopoietic stem cells, Innate immunity, Parasitology

## Abstract

The microbiome provides resistance to infection. However, the underlying mechanisms are poorly understood. We demonstrate that colonization with the intestinal bacterium *Clostridium scindens* protects from *Entamoeba histolytica* colitis via innate immunity. Introduction of *C*. *scindens* into the gut microbiota epigenetically altered and expanded bone marrow granulocyte-monocyte progenitors (GMPs) and resulted in increased intestinal neutrophils with subsequent challenge with *E*. *histolytica*. Introduction of *C*. *scindens* alone was sufficient to expand GMPs in gnotobiotic mice. Adoptive transfer of bone marrow from *C*. *scindens*–colonized mice into naive mice protected against amebic colitis and increased intestinal neutrophils. Children without *E*. *histolytica* diarrhea also had a higher abundance of Lachnoclostridia. Lachnoclostridia *C*. *scindens* can metabolize the bile salt cholate, so we measured deoxycholate and discovered that it was increased in the sera of *C*. *scindens*–colonized specific pathogen–free and gnotobiotic mice, as well as in children protected from amebiasis. Administration of deoxycholate alone increased GMPs and provided protection from amebiasis. We elucidated a mechanism by which *C*. *scindens* and the microbially metabolized bile salt deoxycholic acid alter hematopoietic precursors and provide innate protection from later infection with *E*. *histolytica*.

## Introduction

Commensal intestinal bacteria may protect from infection (1, 2) by modulating bone marrow production of innate immune effector cells, including neutrophils and inflammatory macrophages (3–5). The host metabolome is influenced by the composition of the commensal gut microbiome and is implicated in communicating and directing the development of innate immunity, to some extent, via bile acids (6). Primary bile acids produced by the host and secondary bile acids metabolized by the intestinal microbiota (e.g., deoxycholic and lithocholic acid) can act as signaling molecules, much like host damage-associated molecular pattern molecules (DAMPs) (6). Bile acids within the intestine may protect from intestinal pathogen infection (7). Bile acid receptors are expressed in many cells implicated in innate immunity, are present in the myeloid lineage, and may impact expansion of these cells (8). Bone marrow also has the ability to recognize bile acids (9, 10). Epigenetic effects may result from signaling via bile acids, including inducing methyltransferase activity (11). This may explain in part how infection with one microorganism alters the inflammatory response to other pathogens, providing innate protection from infection with unrelated pathogens (12–15).

Epigenetic changes, such as H3K27 and H3K4 methylation associated with promoter regions of innate inflammatory genes (16–18), have been implicated as a mechanism for this process. As such, commensal microbial metabolite alteration of H3K27 demethylase expression in innate immune populations might contribute to protection from infection (17, 19). Host DAMPs that can be systemically induced by the microbiota have also been shown to be important in upregulating demethylase expression in myeloid cell lines and mouse bone marrow (12, 20). Collectively, these data suggest a role of serum-soluble mediators, including secondary bile acids, induced by the microbiota in communicating to the bone marrow to influence immunity to infection. We sought here to better understand the mechanism by which protective immunity induced by a metabolic product of the microbiota might occur during infection with a human intestinal pathogen.

## Results and Discussion

**Figure 1:**
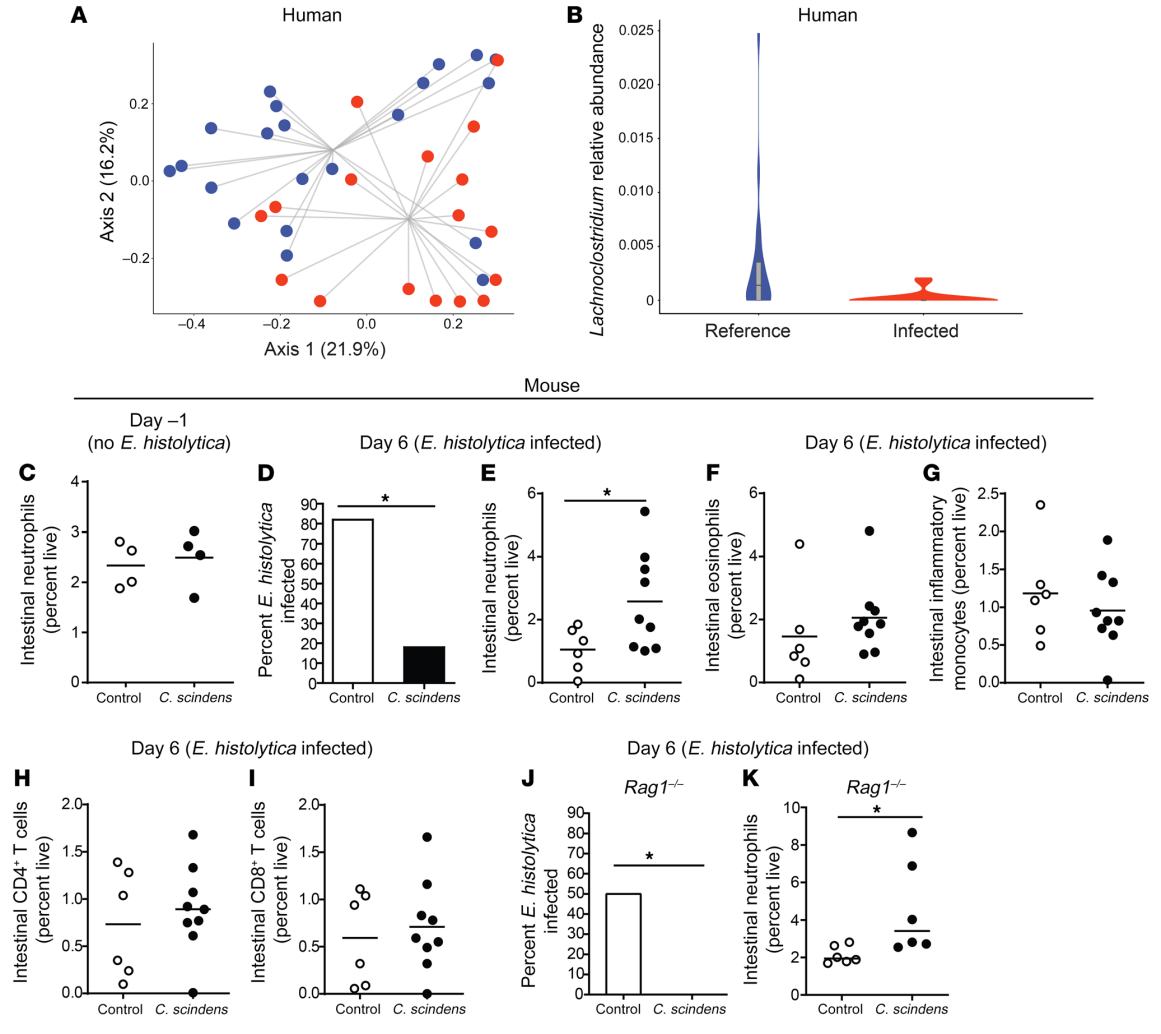
*Lachnoclostridium* are associated with protection from *Entamoeba histolytica* in children, and introduction of Lachnoclostridium *Clostridium scindens* to the gut microbiota provides innate protection from *Entamoeba histolytica* in a murine model. (**A**) Principal coordinates analysis (PCoA) of Bray-Curtis dissimilarities (β-diversity) of fecal microbiota from surveillance reference stool or *E*. *histolytica–*infected children was performed. The groups are significantly different by PERMANOVA: *P* < 0.05. (**B**) Relative abundance of the genus *Lachnoclostridium* from samples described in **A**. The groups are significantly different by Wilcoxon’s rank-sum test with continuity correction: *P* < 0.05; *n* = 20 children per condition. CBA/J mice (**C**–**I**) or C57BL/6 *Rag1^–/–^* mice (**J** and **K**) were colonized with bile acid 7α–dehydroxylating bacteria *C*. *scindens* (ATCC 35704) over 3 weeks before intracecal infection with *E*. *histolytica*. (**C**) Gut neutrophil infiltration was determined before amoeba infection via flow cytometry. (**D** and **J**) Percentage of mice infected with *Entamoeba* on day 6 following infection was determined via cecal culture in trophozoite culture media. (**E**–**I** and **K**) Gut immune cell infiltration was determined via flow cytometry. *P < 0.05 by Student’s *t* test (**C**, **E**–**I**, and **K**) or Mann-Whitney *U* test (**D** and **J**). Horizontal bars indicate the mean. *n* = 4–9 mice per group.

Previous work suggested that murine commensals influence the inflammatory capacity of marrow-derived cells (12, 13). We hypothesized that components of the human gut microbiota might alter bone marrow hematopoiesis to confer protection against unrelated pathogens such as *Entamoeba histolytica* (21, 22). To explore this possibility, we first tested for human commensals associated with protection from amebiasis. Principal coordinates analysis of β-diversity indicated that the microbiota of children with *E*. *histolytica* diarrhea differed significantly (Figure 1A), with a decrease in the relative abundance of the genus *Lachnoclostridium* (Figure 1B). Some *Lachnoclostridium* are known to alter the metabolome, including the bile acid pool of the intestine (7, 23, 24). We hypothesized that these bacteria provide protection from *Entamoeba*. To test this hypothesis we introduced a member of the *Lachnoclostridium* genus, the human commensal bacteria *Clostridium scindens* (24), into the gut microbiome of susceptible CBA/J mice (25) and challenged them with the parasite *E*. *histolytica*.

*C. scindens* was significantly increased in the microbiota after gavage, and gut community structure was also altered (Supplemental Figure 5, A and B; supplemental material available online with this article; https://doi.org/10.1172/JCI133605DS1). Introduction of *C*. *scindens* to the gut microbiome provided protection from *E*. *histolytica* (Figure 1, D and J, Supplemental Figure 4B, and Supplemental Figure 5E) and this protection was associated with increased intestinal neutrophil infiltration (Figure 1, E and K, and Supplemental Figure 3, A and B). This increase in gut neutrophils only occurred with *Entamoeba* infection (Figure 1, C and E). There was no significant difference in intestinal CD4^+^ and CD8^+^ T cells, eosinophils, or inflammatory monocytes (Figure 1, F–I) in *C*. *scindens*–colonized mice.

Myeloid cell expansion may be influenced by cytokine production by CD8^+^ T cells (26) or intestinal Treg cells (27). Contribution of the acquired immune system to *C*. *scindens–*mediated protection was tested by using *Rag1^–/–^* mice, which lack B and T cells. *Rag1^–/–^* mice were also protected from *E*. *histolytica* when colonized with *C*. *scindens* (Figure 1, J and K), indicating that protection did not require the acquired immune system.

**Figure 2:**
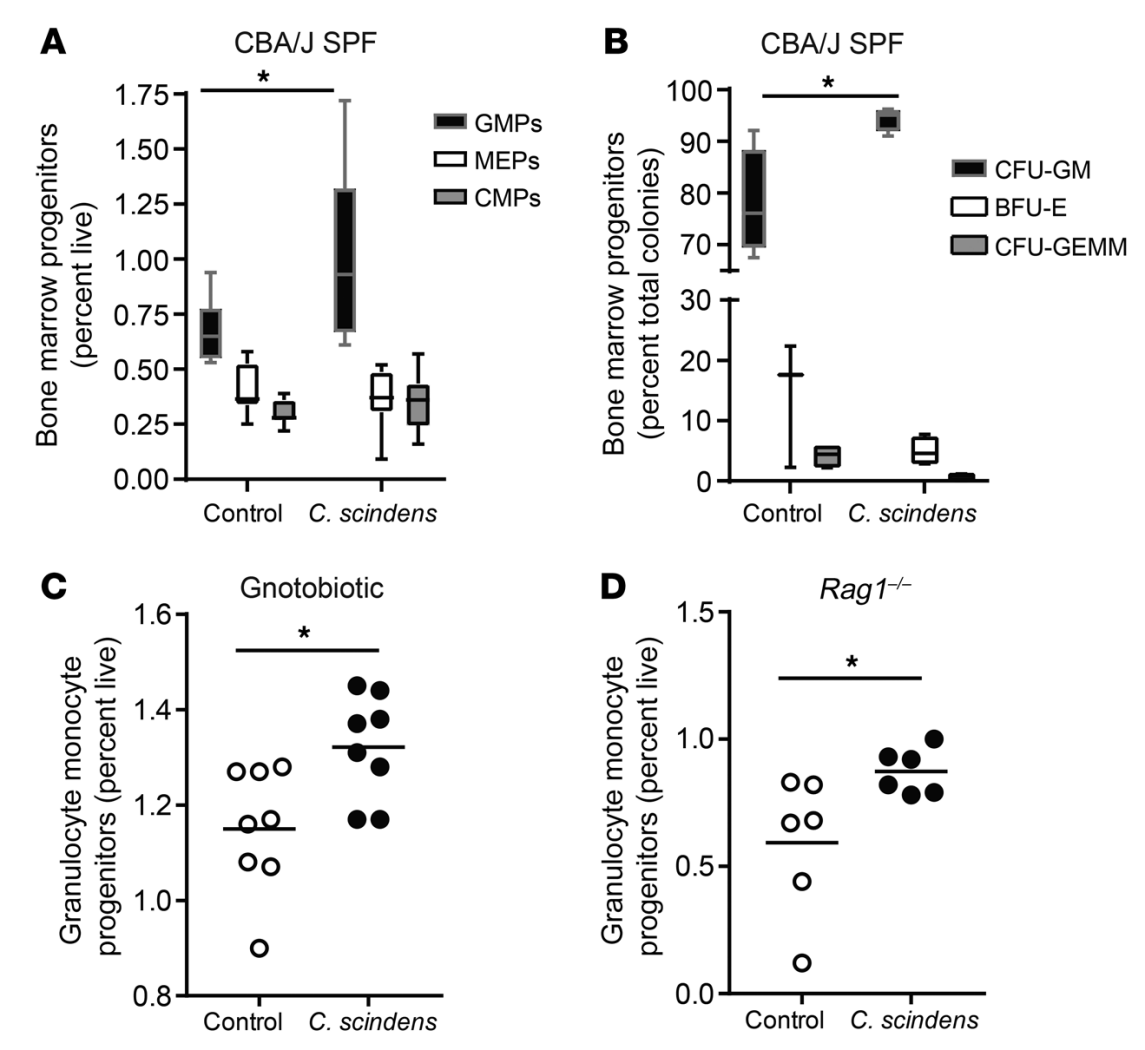
Intestinal colonization with *C.* *scindens* expands bone marrow granulocyte-monocyte progenitors. (**A** and **B**) CBA/J, SPF, (**C**) gnotobiotic C57BL/6, or (**D**) SPF C57BL/6 *Rag1^–/–^* mice were colonized with bile acid 7α–dehydroxylating bacteria *C*. *scindens* (ATCC 35704). (**A**, **C**, and **D**) Flow cytometry and (**B**) colony-forming assays were used to determine composition of marrow hematopoietic precursors in *C*. *scindens*–colonized CBA/J or *Rag1^–/–^* mice. Common myeloid progenitors (CMPs) are Lin^–^c-Kit^+^Sca-1^–^CD34^+^FcgRII/III^int^. Granulocyte-monocyte progenitors (GMPs) are Lin^–^c-Kit^+^Sca-1^–^CD34^+^FcgRII/III^hi^. Megakaryocyte-erythroid progenitors (MEPs) are Lin^–^c-Kit^+^Sca-1^–^CD34^–^FcgRII/III^–^. Colony formation in **B** was assayed for burst-forming unit–erythroid (BFU-E), colony-forming unit–granulocyte/monocyte (CFU-GM), and CFU granulocyte/erythrocyte/monocyte/megakaryocyte (CFU-GEMM). *P < 0.05 by Student’s *t* test. Horizontal bars indicate the mean and whiskers were plotted via Tukey’s method in GraphPad Prism software. *n* = 6–8 mice per group.

The increase in gut neutrophils in response to *Entamoeba* infection in *C*. *scindens*–colonized mice suggested that *C*. *scindens* may have altered innate bone marrow populations that give rise to neutrophils. Therefore, we examined hematopoietic progenitors in *C*. *scindens*–colonized specific pathogen–free (SPF) mice (Figure 2, A and B), SPF *Rag1^–/–^* mice (Figure 2D), and *C*. *scindens* gnotobiotic mice and germ-free controls (Figure 2C). Intestinal colonization with *C*. *scindens* increased bone marrow granulocyte progenitor cells (Figure 2). Expansion of granulocyte-monocyte progenitors (GMPs) mediated by *C*. *scindens* occurred in the absence of T cells (Figure 2D) and colonization with *C*. *scindens* alone was sufficient to increase marrow GMPs, as demonstrated by our study in gnotobiotic animals (Figure 2C). This suggested that innate immune cells underlie the observed *C*. *scindens*–mediated changes in hematopoiesis and protection from *Entamoeba*. The increase in intestinal neutrophils in *C*. *scindens–*colonized mice only occurred following *Entamoeba* challenge. This suggested there may be homeostatic changes in pathways in GMPs important in neutrophil production (5).

To explore this possibility, we examined transcriptional and epigenetic changes in marrow GMPs from *C*. *scindens*–colonized mice. Gene enrichment analysis of RNA sequencing data suggested that genes associated with covalent modification of the histone H3 tail, such as the demethylase JMJD3, were enriched in mice exposed to *C*. *scindens* (Supplemental Figure 3F). This analysis also indicated enrichment of genes associated with CCAAT/enhancer-binding proteins, known to be important for GMP and neutrophil differentiation and expansion (refs. 30, 31 and Supplemental Figure 3F). Quantitative PCR of sorted marrow GMPs confirmed that significant changes in expression occurred in JMJD3 (Supplemental Figure 3, C–E, G, J, and K) and 2 CCAAT/enhancer-binding protein genes important in granulopoiesis (28, 29), C/EBPA (Supplemental Figure 3H) and C/EBPB (Supplemental Figure 3I). Therefore, we examined H3K4me3 and H3K27me3 occupancy in the promoter regions of C/EBPA and C/EBPB in sorted GMPs. The repressive mark H3K27me3 was decreased in the promoter of C/EBPA in *C*. *scindens*–colonized mice (Supplemental Figure 3L), while the activating mark H3K4me3 (Supplemental Figure 3M) was increased in the promoter of C/EBPB in *C*. *scindens*–colonized mice. Therefore, bone marrow epigenetic alteration occurred with gut colonization by *C*. *scindens*. This suggested bone marrow changes might underlie gut immunity to *Entamoeba* in colonized mice. To explore this possibility we used adoptive marrow transplants.

**Figure 3:**
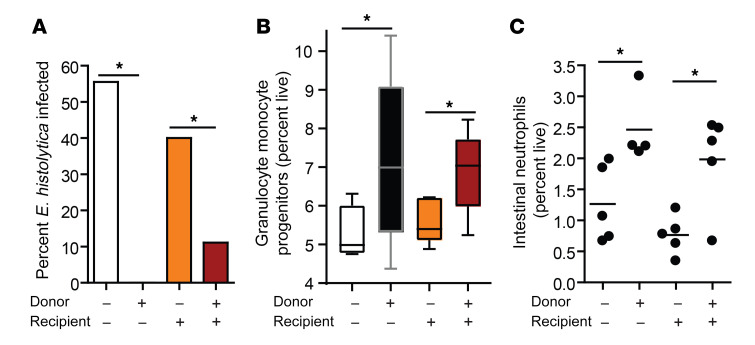
Bone marrow from *C.* *scindens*–colonized donors is sufficient to provide protection from *Entamoeba* in *C. scindens*–naive mice. CBA/J mice colonized with *C*. *scindens* (+) or not (–) were lethally irradiated and given whole marrow from *C*. *scindens* (+) or *C*. *scindens* (–) donors and then allowed to recover for 7 weeks before *Entamoeba* challenge. (**A**) Protection from amoebic colitis, (**B**) change in marrow GMPs, and (**C**) gut neutrophil infiltration were determined at 8 weeks after BMT. *P < 0.05 by Mann-Whitney *U* test (**A**) or 1-way ANOVA with Tukey’s post hoc test (**B** and **C**). Horizontal bars indicate the mean and whiskers were plotted via Tukey’s method in GraphPad Prism software. *n* = 4–8 mice per group.

Adoptive transfer of bone marrow from *C*. *scindens*–colonized mice into mice not previously exposed to *C*. *scindens* was sufficient to provide protection from *E*. *histolytica* (Figure 3A) as well as recapitulate the increase in marrow GMPs (Figure 3B) and intestinal neutrophils (Figure 3C). In contrast, previous epithelial exposure to *C*. *scindens* was not sufficient to provide protection from amoeba in irradiated mice (Figure 3A). We also noted an overall increase in GMPs in the mice after bone marrow transplant; however, this increase was controlled across all groups and was likely a response to irradiation (30). We concluded that alterations in radio-sensitive marrow hematopoietic cells caused by gut exposure to C. *scindens* were sufficient to confer protection to a later *E*. *histolytica* challenge.

**Figure 4:**
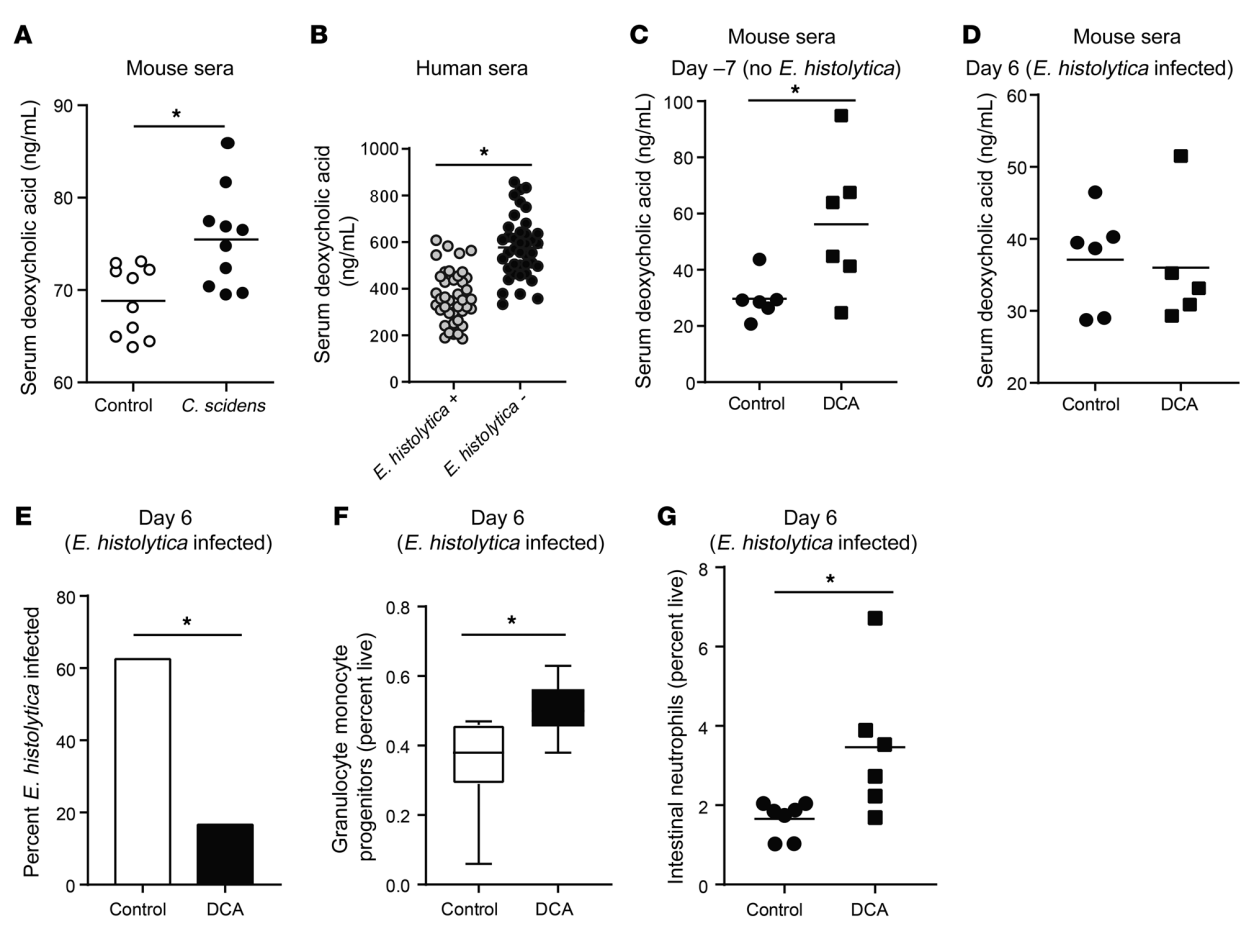
*C.scindens* colonization increases serum deoxycholic acid (DCA), and administration of DCA expanded marrow GMPs and intestinal neutrophils and protected from amoebic colitis. (**A**) CBA/J mice were colonized with *C*. *scindens* over 3 weeks via gavage and serum DCA was measured at 10 weeks of age in control BHI media–gavaged mice and *C*. *scindens*–gavaged mice. (**B**) Serum DCA was measured via ELISA in 2-year-old children in Bangladesh free of (–) or infected with (+) *E*. *histolytica* within 6 months of the blood draw. *n* = 40 children per condition. (**C**–**G**) Mice were administered DCA or PBS intravenously 3 times a week for 2 weeks and then challenged with *E*. *histolytica*. Serum DCA was measured at the end of week 1 (day –7) (**C**) and at the end of the experiment (day 6) (**D**). (**E**) *E*. *histolytica* infection, (**F**) change in marrow GMPs, and (**G**) intestinal neutrophils were measured at the end of the experiment. *P < 0.05 by Student’s *t* test (**A**–**D**, **F**, and **G**) or Mann-Whitney *U* test (**E**). *n* = 6–8 mice per group.

We next explored how intestinal colonization with *C*. *scindens* could be altering GMPs in the bone marrow. *C*. *scindens* is known to be capable of 7α-dehydroxylation of bile acids in the intestine (24). Gavage and colonization with another human mucosal anaerobic bacterium lacking 7α-dehydroxylation activity did not induce protection from *Entamoeba* (Supplemental Figure 5, C–E). Colonization of mice with *C*. *scindens* was sufficient to increase serum levels of the secondary bile acid deoxycholic acid (DCA, a product of 7α-dehydroxylation of cholic acid) in SPF and in gnotobiotic mice (Figure 4A and Supplemental Figure 2, A and B). However, absolute levels of DCA were lower in gnotobiotic mice in both groups than in SPF. These lower absolute levels are perhaps due to the lack of other members of the microbiota producing products upstream of 7α-dehydroxylation (31). DCA was also increased in children (from 2 independent cohorts) protected from *E*. *histolytica* (Figure 4B and Supplemental Figure 1, A and B). We concluded that DCA in plasma was positively correlated with protection from *Entamoeba* in the mouse model of amebic colitis and in children. Future studies may examine these effects in adult patients.

To test if transient elevation of serum DCA was sufficient to mediate protection from *Entamoeba* we administered the bile salt intravenously. Administration of DCA before *Entamoeba* infection increased serum levels of deoxycholate (Figure 4, C and D) and provided protection from infection (Figure 4E). Treatment of mice with DCA was not associated with elevated markers of liver damage or intestinal inflammation before *Entamoeba* infection (Supplemental Figure 6). Protection from *Entamoeba* was associated with increased marrow GMPs and gut neutrophils (Figure 4, F and G). Experimental elevation of serum DCA increased expression of the epigenetic mediator JMJD3 in sorted marrow GMPs (Supplemental Figure 3K). We concluded that DCA was sufficient to recapitulate the changes in GMPs and protection from *Entamoeba* afforded by *C*. *scindens*. This work provides evidence that *C*. *scindens* and the microbially metabolized bile salt DCA are sufficient to alter hematopoietic precursors and provide innate protection from later infection. These studies, however, do not rule out the contribution of other bile acids and metabolites to gut-to–bone marrow communication.

Deoxycholate-mediated protection from *E*. *histolytica* was associated with increased marrow GMPs and intestinal neutrophils, as seen with *C*. *scindens*. We next explored pathways by which deoxycholate or *C*. *scindens* might increase GMPs. Owing to the epigenetic changes observed (Supplemental Figure 3, L and M), persistent nature of immunity to *E*. *histolytica* following bone marrow transplant in the absence of colonization with the commensal (Figure 3), and upregulation of JMJD3 in sorted marrow from *C*. *scindens*–colonized or DCA-treated mice (Supplemental Figure 3, G, J, and K), we examined the role of JMJD3 activity during *C*. *scindens* colonization in protection from *Entamoeba* infection. Treatment with an inhibitor of JMJD3 during *C*. *scindens* colonization abrogated bone marrow GMP expansion (Supplemental Figure 4A) as well as induction of intestinal neutrophils and protection from *E*. *histolytica* (Supplemental Figure 4, B and C). This suggests H3K27 demethylase activity may contribute to gut-to-marrow communication by *C*. *scindens*. JMJD3 is an H3K27me3 demethylase (17); however, we also observed changes in H3K4me3 in the promoter region of C/EBPB. JMJD3 has recently been shown to impact H3K4me3 levels in human acute myeloid leukemia (AML) cells (32). This may not fully explain the epigenetic changes in our model, and other epigenetic mediators, including other non-methyl modifications, might influence gut microbiota–mediated communication with the bone marrow.

The results presented here suggest a model whereby gut colonization with *C*. *scindens* increases serum deoxycholate that then acts on the marrow to increase transcription of genes that support GMP expansion, such as CCAAT/enhancer-binding proteins C/EBPA and C/EBPB. Then, when a different challenge occurs at a mucosal site (in this case infection with *E*. *histolytica*), a more robust neutrophil response results.

Future studies will examine the precise mechanisms by which *C*. *scindens* colonization alters bone marrow hematopoiesis, which are not fully elucidated by these studies. However, this work yields understanding of how changes in the gut microbiome can result in antigen-nonspecific protection from *E*. *histolytica* infection. This heightened inflammatory response may have implications for other infectious diseases, and potentially other mucosal sites in the body such as the lung. Therefore, the impact of the work extends beyond infectious disease to fundamental mechanisms of gut-to–bone marrow communication by commensal bacteria. These studies may help in development of microbiome-targeted treatments that modulate the severity of immune and inflammatory diseases by altering bone marrow production of inflammatory cells.

## Methods

More information is available in the supplemental methods. Sequencing data have been deposited in NCBI’s Gene Expression Omnibus (GEO) repository under accession number GSE121503, the SRA under accession number PRJNA503904, and under SRA and linked via the dbGaP accession number phs001478.v1.p1.

### Statistics.

All *P* values of less than 0.05 were considered significant. All Student’s *t* tests were 2 tailed. In the box-and-whisker plots in Figures 2 and 3, horizontal bars indicate the mean and the whiskers were plotted via Tukey’s method in GraphPad Prism software as follows. The interquartile distance was calculated as the difference between the 25th and 75th percentiles and is referred to as the IQR. If the 75th percentile plus 1.5 × IQR was greater than (or equal to) the largest value in the data set, then the upper whisker extends to the largest value. Otherwise, the upper whisker ends at the largest value less than the sum of the 75th percentile plus 1.5 × IQR, and any values that were greater than this were plotted as individual points. If the 25th percentile minus 1.5 × IQR was less than the smallest value in the data set, then the lower whisker extends to the smallest value. Otherwise, the lower whisker ends at the lowest value greater than the 25th percentile minus 1.5 × IQR, and any values that were greater than this were plotted as individual points. Additional statistical method details are available in the supplemental methods.

### Study approval.

Design of the human cohort studies has been described (33, 34) and all studies were approved by the Research and Ethical Review Committees of the International Centre for Diarrhoeal Disease Research, Bangladesh (icddr,b) and the Institutional Review Boards of the University of Virginia. All animal procedures were approved by the Institutional Animal Care and Use Committee of the University of Virginia. All experiments were performed according to provisions of the Animal Welfare Act of 1996 (§ 89.544).

## Author contributions

SLB, JLL, JU, DNO, KW, MS, MS, NG, BAT, and BM performed experiments. SLB, JLL, DTA, ST, DNO, NG, JZM, ZP, BM, CG, and BAT analyzed the data. WAP, SLB, JS, RH, and JZM supervised the experiments and data analysis. SLB and WAP developed the theoretical framework. All authors discussed the results and contributed to the preparation of the manuscript.

## Supplementary Material

Supplemental data

## Version 1 05/05/2020

In-Press Preview

## Version 2 06/22/2020

Electronic publication

## Version 3 08/03/2020

Print issue publication
