# Supplementary material for: Gut microbiome communication with bone marrow regulates susceptibility to amebiasis
Source: J Clin Invest. 2020 Jun 22;130(8):4019–24. doi: 10.1172/JCI133605 (PMC7410058; doi:10.1172/JCI133605)
Supplement: Supplemental data [file jci-130-133605-s005.pdf]

Supplementary Materials for

Title: Gut microbiome communication with bone marrow regulates susceptibility to amebiasis.

Figures S1 to S6

Materials and Methods

Supplemental References

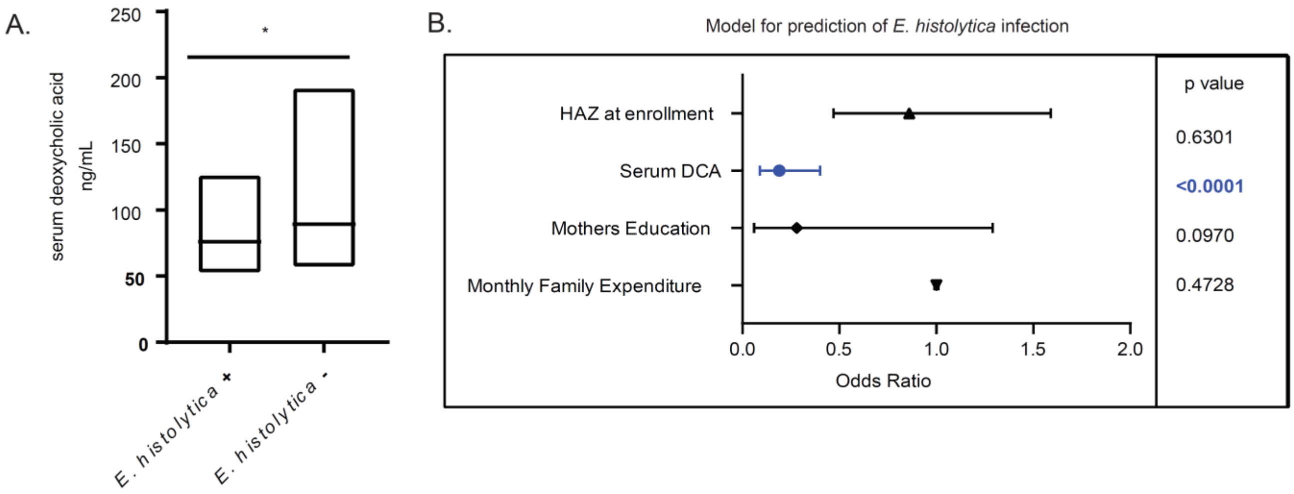

Figure S1. Serum DCA predicts intestinal *E. histolytica* infection in two childhood cohorts in Bangladesh.

(A) Serum DCA was measured via ELISA in 2 year old children in Bangladesh that were free of (-) or infected with (+) *E. histolytica* within 6 months of the blood draw**.** Sera from this cohort underwent an additional freeze and thaw cycle compared to the second cohort in Figure 4 and in **B**, below. *= p<0.05, Student's t-test, N= 40 children per condition. **(B)** The association of serum DCA with intestinal *E. histolytica* infection was evaluated in logistic regression in 2 year old children in a second birth cohort in Bangladesh (PROVIDE), adjusting for Height versus Age Z score (HAZ) at enrollment, monthly family expenditure, and mother's education. **(A)** *= p<0.05, Student's t-test **(B)**, Odds ratio, P value, and 95% confidence interval are shown. **(A, B)** N= 40 children per condition.

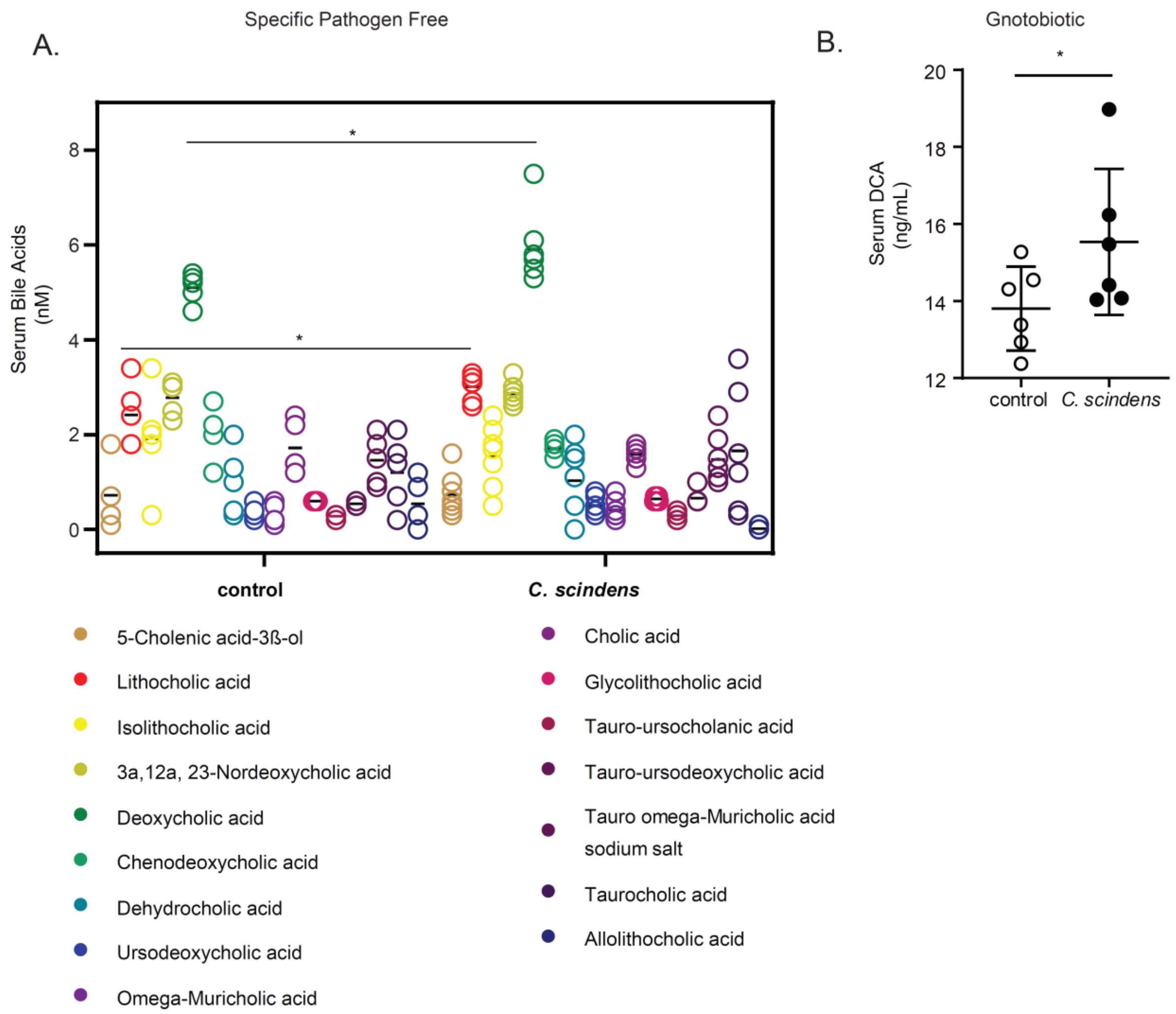

Figure S2. Serum bile acid profile of *Clostridium scindens* colonized mice. (A) Specific pathogen free (SPF) CBA/J mice or **(B)** gnotobiotic C57BL/6 mice were colonized with *C. scindens* (ATCC® 35704), a bacterium capable of the 7α-dehydroxylation of bile acids. **(A)** Serum bile acids were measured using ultra-performance liquid chromatography-mass spectrometry in control and *C. scindens* gavaged animals**.** *= $p<0.05$, Fishers LSD. N =6-7 mice per group **(B)** Serum DCA was measured using ELISA in *C. scindens* gavaged and control gnotobiotic animals. *= $p<0.05$, students T test, bars indicate mean and SEM, N=6-8 mice per group.

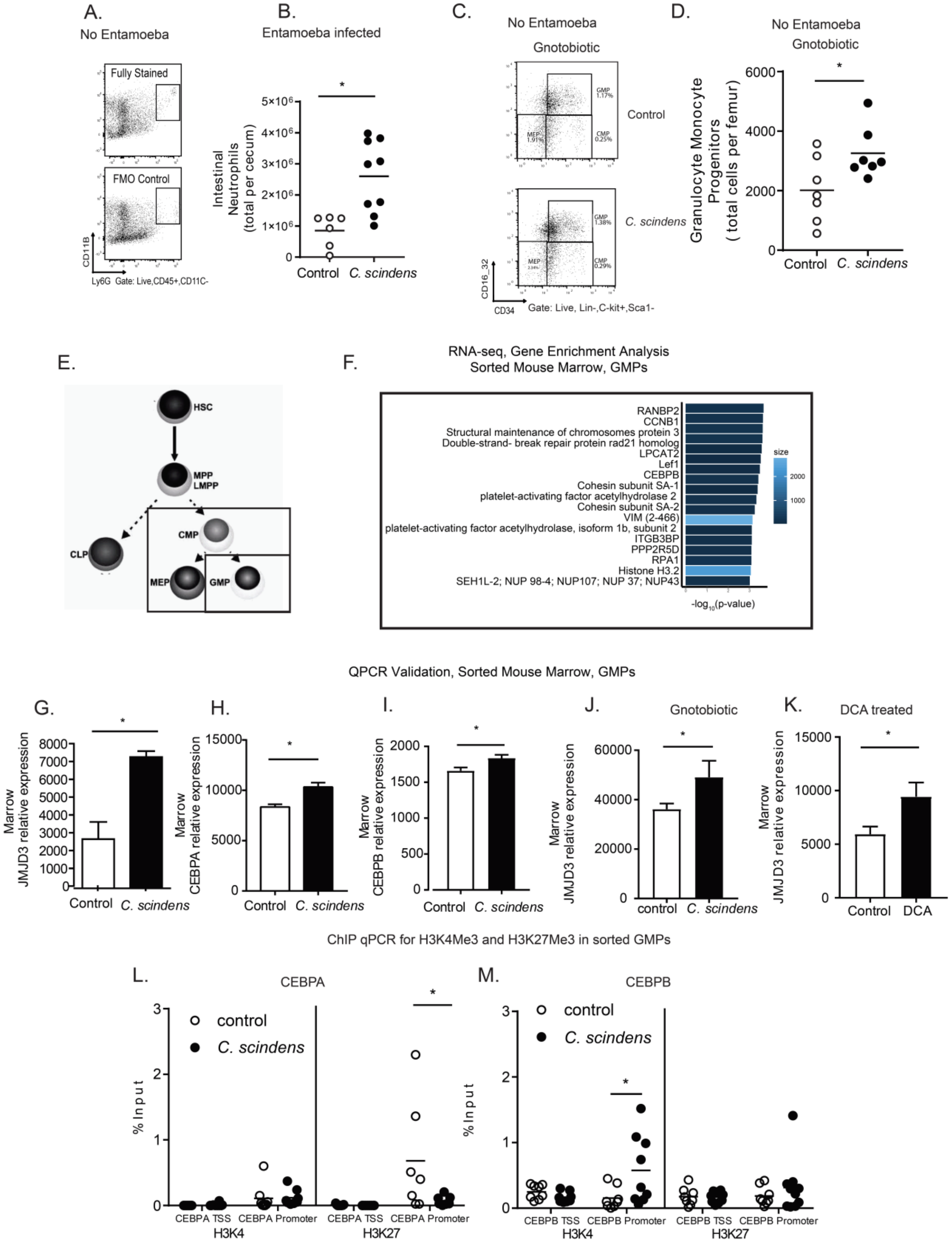
A.
No Entamoeba
Fully Stained
FMO Control
CD11B
Ly6G Gate: Live,CD45+,CD11C-
B.
Entamoeba infected
Intestinal Neutrophils (total per cecum)
Control
C. scindens
C.
No Entamoeba
Gnotobiotic
Control
C. scindens
GMP
CMP
MEP
CD16_32
CD34
Gate: Live, Lin-,C-kit+,Sca1-
D.
No Entamoeba
Gnotobiotic
Granulocyte Monocyte Progenitors (total cells per femur)
Control
C. scindens
E.
HSC
MPP
LMPP
CMP
CLP
MEP
GMP
F.
RNA-seq, Gene Enrichment Analysis
Sorted Mouse Marrow, GMPs
RANBP2
CCNB1
Structural maintenance of chromosomes protein 3
Double-strand- break repair protein rad21 homolog
LPCAT2
Lef1
CEBPB
Cohesin subunit SA-1
platelet-activating factor acetylhydrolase 2
Cohesin subunit SA-2
VIM (2-466)
platelet-activating factor acetylhydrolase, isoform 1b, subunit 2
ITGB3BP
PPP2R5D
RPA1
Histone H3.2
SEH1L-2; NUP 98-4; NUP107; NUP 37; NUP43
size
-log10(p-value)
QPCR Validation, Sorted Mouse Marrow, GMPs
G.
Marrow JMJD3 relative expression
Control
C. scindens
H.
Marrow CEBPA relative expression
Control
C. scindens
I.
Marrow CEBPB relative expression
Control
C. scindens
J.
Gnotobiotic
Marrow JMJD3 relative expression
control
C. scindens
K.
DCA treated
Marrow JMJD3 relative expression
Control
DCA
ChIP qPCR for H3K4Me3 and H3K27Me3 in sorted GMPs
L.
CEBPA
control
C. scindens
% Input
CEBPA TSS
CEBPA Promoter
H3K4
H3K27
M.
CEBPB
control
C. scindens
% Input
CEBPB TSS
CEBPB Promoter
H3K4
H3K27

Figure S3. Intestinal colonization with *Clostridium scindens* increases number of intestinal neutrophils and bone marrow GMPs, epigenetically alters GMPs, and increases expression of H3K27 demethylase JMJD3 and granulopoiesis promoting transcription factors CEBPA and CEBPB in GMPs. Representative gating and number of cells per organ is shown for **(A)** intestinal neutrophils in CBA/J *C. scindens* colonized and uninfected mice, **(B)** control and CBA/J *C. scindens* colonized mice following Entamoeba infection and **(C,D)** bone marrow GMPs in gnotobiotic C57BL/6 mice. **(E)** Bone marrow was sorted into CMP, GMP, MEP and CLP from SPF CBA/J **(F-I, K, L, M)**, or C57BL/6 Gnotobiotic mice **(J)** that were colonized with *Clostridium scindens* or treated with DCA **(K). (F)** RNA-seq Gene Enrichment Analysis (GEA) and **(G-K)** qPCR was performed to validate enriched genes from RNA-seq GEA on cDNA prepared from RNA isolated from sorted GMPs. **(F)** Gene set enrichment results obtained using ConsensusPathDB. The plot shows the most over-represented functional gene clusters associated with the top 50% of genes ranked by unadjusted P value and the size indicates number of genes in each cluster**.** CEBPB and Histone H3.2 gene sets were identified as enriched functional networks using this unbiased approach. CEBPA and CEBPB are included in the CEBPB gene set and JMJD3 (KDM6B) was included in the H3.2 gene set (**F)** Expression of noted genes was measured via QPCR and normalized to a housekeeping gene (S14) in **(G-K)**. JMJD3 expression was measured in **(J)** gnotobiotic mice and **(K)** CBA/j mice treated with DCA. **(L, M)** ChIP for H3K4me3 and H3K27me3 was performed followed by qPCR for the transcription start site (TSS) and promoter for **(L)** CEBPA and **(M)** CEBPB on sorted GMPs from mice colonized with *C. scindens* or control mice. *= $p<0.05$, **(A-K)**, Student's t-test, **(L, M)** One Way ANOVA with Tukey posttest. N =6-12 mice per group

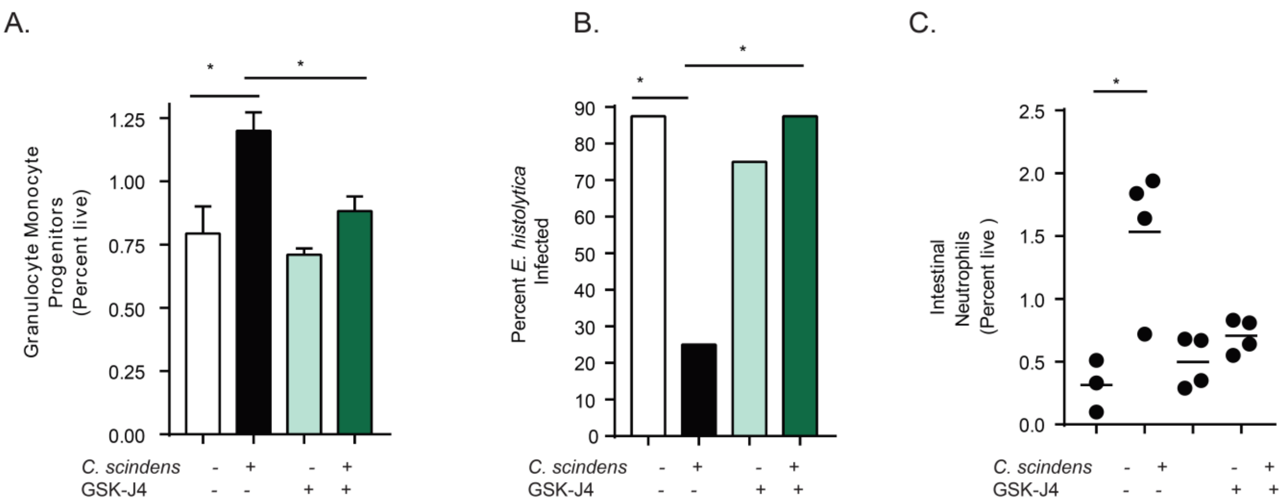

Figure S4. Blockade of H3K27 demethylase JMJD3 during *C. scindens* colonization abrogates marrow GMP expansion and intestinal protection from *E. histolytica.* CBA/J mice were treated with an inhibitor of the epigenetic mediator JMJD3 (GSK-J4) (**-**, **+**) before and during *C. scindens* colonization (**-**, **+**) but prior to infection with ameba. **(A)** Bone marrow progenitor populations, **(B)** percent infectivity and **(C)** intestinal neutrophils were analyzed via culture and flow cytometry. *= p<0.05, **(A, C)** Student's t-test, **(B)** Mann–Whitney U test, one way ANOVA with Tukey posttest. N =4-12 mice per group.

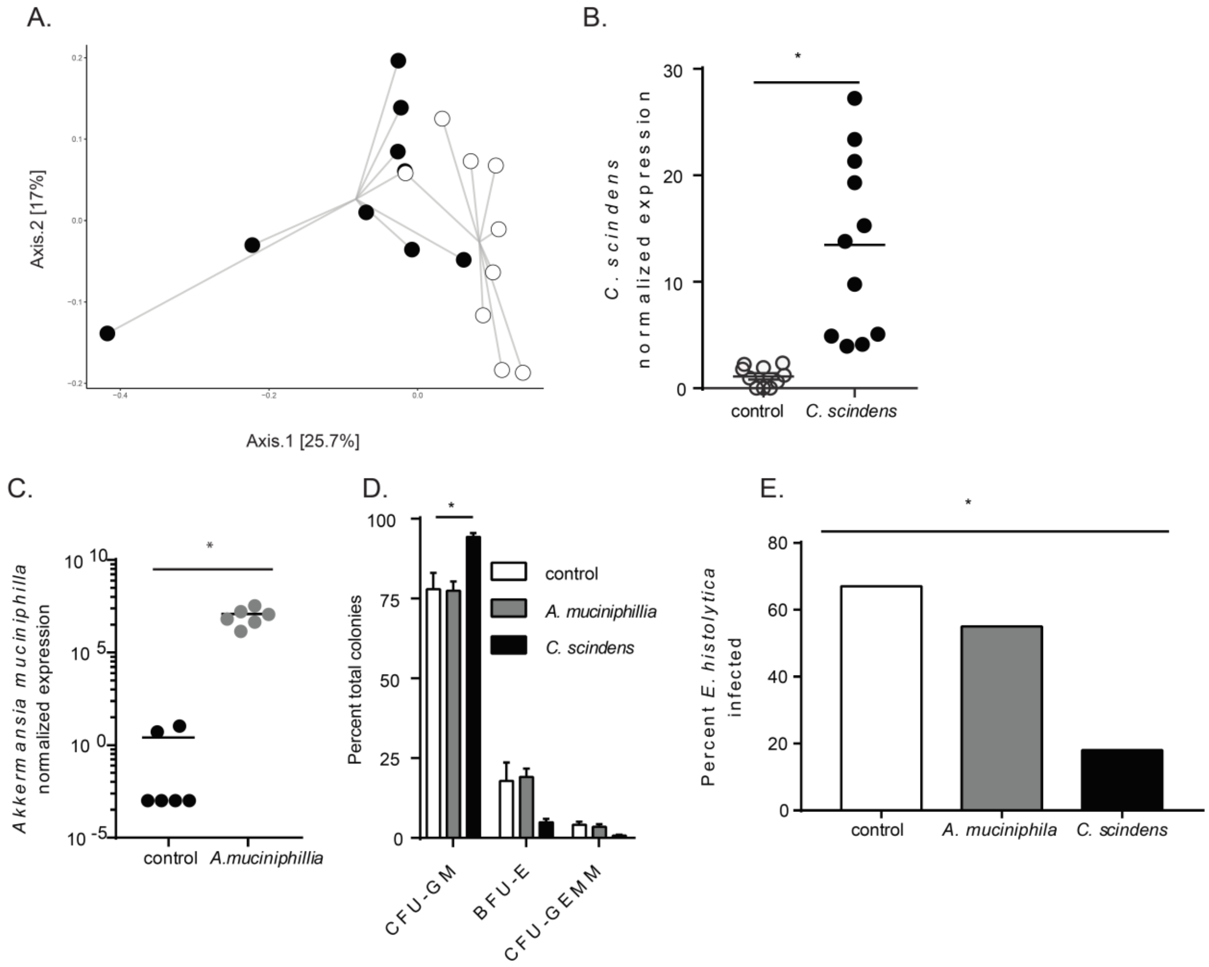
A.
Axis.2 [17%]
Axis.1 [25.7%]
B.
*
C. scindens
normalized expression
30
20
10
0
control
C. scindens
C.
*
Akkermansia muciniphilla
normalized expression
10 10
10 5
10 0
10 -5
control
A.muciniphillia
D.
*
100
75
50
25
0
Percent total colonies
control
A. muciniphillia
C. scindens
CFU-GM
BFU-E
CFU-GEMM
E.
*
80
60
40
20
0
Percent E. histolytica infected
control
A. muciniphila
C. scindens

Figure S5. Introduction of *Clostridium scindens* to the gut microbiota alters community structure in mice and introduction of human commensal *Akkermansia muciniphila* to the gut microbiota does not increase marrow GMPs or provide significant protection from *E. histolytica.* CBA/J mice were colonized with bile acid 7α-dehydroxylating bacteria *C. scindens* (ATCC® 35704) over three weeks. **(A)** Composition of the cecal microbiota community structure was determined by sequencing of V4 region of the 16S rRNA gene. PCoA of Bray-Curtis dissimilarities is shown, the groups are significantly different by PERMANOVA, p = 0.002. **(B)** Relative expression of *C. scindens* baiCD stereo-specific 7alpha/7beta-hydroxy-3-oxo-delta4-cholenoic acid oxidoreductase normalized to total eubacterial 16S rRNA was determined via qPCR**.** *= p<0.05. N =9-12 mice per group. CBA/J mice were colonized with *Akkermansia muciniphila* (ATCC ®BAA-835) or *C. scindens* (ATCC® 35704) over three weeks. **(C)** Relative expression of *Akkermansia muciniphila* 16s rRNA normalized to total eubacterial 16S rRNA was determined via qPCR. **(D)** Composition of marrow hematopoietic precursors was determined via colony forming assays in mice. **(E)** Percent infectivity with ameba at day six following infection was determined via cecal culture in trophozoite supporting media. *= p<0.05, Student's t-test, bars are mean. N =6 mice per group.

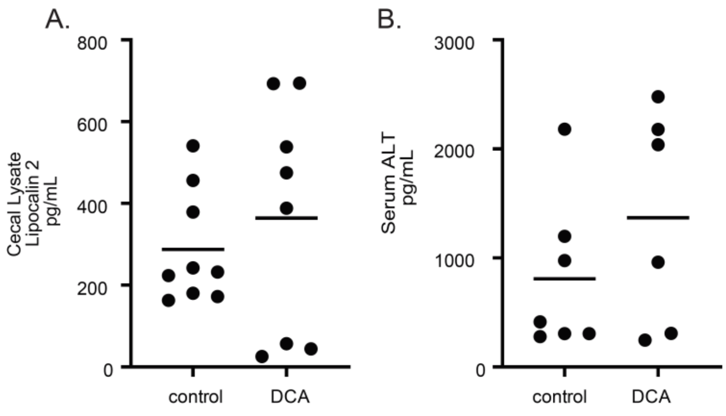

Figure S6. Intravenous treatment of mice with DCA was not associated with elevated markers of liver damage or intestinal inflammation prior to Entamoeba infection. CBA/J mice were treated intravenously via tail vein injection by a trained veterinary technician 3 times a week, over two weeks with 400uL of PBS or 400uL of 0.20mg/mL DCA (Sigma) in PBS per treatment. Mice were healthy during DCA administration and **(A)** no intestinal inflammation as measured via cecal Lipocalin 2 ELISA(R and D, DY1857) or liver damage as measured via **(B)** serum ALT ELISA (Cloud-Clone Corp, SEA207Mu) was observed after treatment.

Methods

Mice

Five week old male CBA/J mice (Jackson Laboratories or RAG 1 KO mice (Jackson Laboratories) were housed in a specific pathogen–free facility in micro isolator cages and provided autoclaved food (Lab diet 5010) and water ad libitum. Specific pathogen free status was monitored quarterly. Quarterly, a sentinel mouse (or rat) was removed from each room and humanely euthanized for serologic evaluation, examination of pelage for fur mites, and examination of cecal contents for pinworms. The serologic assays, conducted in-house using CRL reagents, were MHV, EDIM, GD-7, MVM, MPV, and MNV, (Sendai, PVM, RPV/KRV/H-1, *M. pulmonis*, and SDAV for rats). In the final quarter, a comprehensive serology was run which included the above agents plus K-virus, MCMV, MTV, LCM, Ectromelia, Polyomavirus, Reovirus-3, and mouse adenoviruses (K87 and FL). For Gnotobiotic experiments Germ free C57BL/6 mice (Taconic) were housed in flexible film units (Park Bio) in a facility regularly monitored for germ free status by aerobic and anaerobic culture and the above assays by the UVA Center for Comparative medicine. Cylinders were prepared and supplies and sterile food and water were introduced into the units according to SOP. Germ-free mice from Taconic were introduced into the units. Mice were gavaged once with sterile media or *C. scindens* as in SPF mice below. Aerobic and anaerobic cultures of fecal samples were performed at introduction and once during the 2 week experiment as well as Sanger sequencing of isolated stool DNA using broad range eubacterial primers to confirm mono-association with *C. scindens* and germ free status of control animals. EUB forward 5'-ACTCCTACGGGAGGCAGCAGT-3'EUB reverse 5'-ATTACCGCGGCTGCTGGC-3'. One-week and two weeks post animal placement in the unit, surface cultures were performed to ensure sterility of unit. All contact areas, shipping

containers and transfer apparatus were also cultured to confirm germ free status. All procedures were approved by the Institutional Animal Care and Use Committee of the University of Virginia. All experiments were performed according to provisions of the USA Animal Welfare Act of 1996 (Public Law 89.544). All experiments shown are representative of 2-4 experimental replicates.

Clostridium scindens **colonization**

CBA/J mice (Jackson) were colonized with bile acid 7α-dehydroxylating bacteria *C. scindens* (ATCC® 35704) over three weeks prior to intracecal infection with *E. histolytica* or analysis for gut microbiome community structure, or marrow RNA seq and ChIP . Mice were gavaged with 100ul of overnight culture at an optical density of 1.4 at 600nm or media control (BHI, Anaerobe Systems, AS-872) once per week for specific pathogen free mice over three weeks, and once for C57BL/6 (Taconic) gnotobiotic mice over two weeks.

Intravenous deoxycholate treatment

CBA/J mice were treated intravenously via tail vein injection by a trained veterinary technician 3 times a week, over two weeks with 400uL of PBS or 400uL of 0.20mg/mL DCA (Sigma) in PBS per treatment.

Adoptive Marrow Transplant

Donor and recipient CBA/J mice were colonized with bile acid 7α-dehydroxylating bacteria *C. scindens* (ATCC® 35704) or treated with media controls as above. CBA/J mice colonized with *C. scindens* (+) or not (-) were lethally irradiated, then immediately given whole marrow from *C.*

scindens (+) or *C. scindens* (-) donors then allowed to recover for 7 weeks prior to ameba challenge. Irradiation was performed with 900 Rad in a single dose from a Shepard irradiator, Mark 1 Model 68A Dual, Serial Number 1163 with a Cs-137 source. All irradiation experiments were supervised by a member of the University of Virginia Environmental Health and Safety team. Mice were place on placed on sulfamethoxazole-trimethoprim containing water 3 days prior and 21 days post bone marrow transplant. Experiments shown are representative of 2 experimental replicates.

***E. histolytica* culture and intracecal injection.**

Animal-passaged HM1:IMSS *E. histolytica* trophozoites were cultured from cecal contents of infected mice in complete trypsin-yeast-iron (TYI-33) medium supplemented with Diamond Vitamin mixture (JRH Biosciences), 100 U/ml of both penicillin and streptomycin, and 5% heat inactivated bovine serum (Sigma-Aldrich). Prior to injection, trophozoites were grown to log phase, and 3 $x10^6$ parasites were suspended in 100 µL culture media and injected intracecally (6). Data analysis and graphing was performed with Graphpad Prism 8.0. Final figures were modified and arranged in Adobe Illustrator.

JMJD3 Blockade

Mice were treated intraperitoneally with hybridoma grade DMSO in PBS (Sigma) or GSK-J4 in DMSO/PBS (100uL, DMSO/PBS, 25 mg/kg, Cayman Chemical #12074) one day before and during *C. scindens* colonization (once per week) but not during *E. histolytica* infection. Experiments shown are representative of 2 experimental replicates.

Flow cytometry of intestinal cells

Minced intestinal tissue was digested in Liberase TL (0.17 mg/ml Roche) and DNase (0.5 mg/ml, Sigma) for 45 min at 37°C and processed into a single cell suspension following washing with a buffer containing EDTA. $1x10^6$ cells per mouse were stained with antibodies from Bio Legend, and BD Biosciences. Flow cytometric analysis was performed on an LSR Fortessa (BD Biosciences) and data analyzed via FlowJo (Tree Star Inc.). All gates were set based on fluorescence minus one (FMO) controls. Further data analysis and graphing was performed with Graphpad Prism 7.0. Final figures were modified and arranged in Adobe Illustrator. Experiments shown are representative of 2-4 experimental replicates.

Antibodies

Name	Flourochrome	Clone	Supplier	Catalog number
CD11c	BV421	N418	BioLegend	117330
CD4	BV605	RM4-5	BioLegend	100547
Ly6c	FITC	HK1.4	BioLegend	128005
CD3e	PerCp Cy5.5	145-2C11	BioLegend	100328
SIGLEC F	PE	E50-2440	BD Biosciences	BD552126
Ly6G	PE Cy7	1A8	BioLegend	127618
CD11b	APC	M1/70	BioLegend	101212
CD8a	AF700	53-6.7	Thermo Fisher	56-0081-82
CD45	APC Cy7	30-F11	BioLegend	103116
Live/Dead	Zombie Aqua		BioLegend	423102

Bone marrow flow cytometry and cell sorting

Bone marrow cells were isolated from femur, fibula and tibia of mice by centrifugation in custom made microcentrifuge tubes composed of a 0.5ml microcentrifuge tube with a hole punched in the bottom nested inside a 1.5mL microscentrifuge tube (VWR). Bone marrow cells were stained with PerCP-Cy5.5-labeled lineage (Lin) markers (TCRb, CD3e,CD49b, B220Gr1, CD11c, CD11b), anti-CD34-Brilliant Violet 421, c-Kit-Brilliant Violet 605, CD127-PE-Cy7,

FcgRII-III (CD16/CD42)-APC-Cy7, and Sca-1-APC, CD150 PE, Live dead staining with Zombie Aqua (Bioledgend) or 7AAD for sort experiments. Flow cytometric analysis was performed on an LSR Fortessa (BD Biosciences) or cells sorted on a Becton Dickinson Influx Cell Sorter into RNA later (Qiagen) or Cryostore CS10 (Stemcell) with 7AAD as a live dead stain . Data analyzed via FlowJo (Tree Star Inc.). All gates were set based on fluorescence minus one (FMO) controls. Common Lymphoid Progenitors (CLP) are Lin-IL-7R+c-Kit^{int}Sca-1^{int}, Common Myeloid Progenitors (CMP) are Lin-c-Kit+Sca-1-CD34+FcgRII-III^{int}; Granulocyte-Monocyte-Progenitors (GMP) are Lin-c-Kit+Sca-1-CD34+FcgRII-III^{hi}; Megakaryocyte–Erythroid Progenitors (MEP) are Lin-c-Kit+Sca-1-CD34-FcgRII-III-. Experiments shown are representative of 2-4 experimental replicates.

Antibodies

Name	Flourochrome	Clone	Supplier	Catalog number
TCRb	PerCP-C5.5	H57-597	BioLegend	109227
CD3e	PerCp Cy5.5	145-2C11	BioLegend	100328
CD49b	PerCP-C5.5	DX5	BioLegend	108915
B220	PerCP-C5.5	RA3-6B2	BioLegend	103235
Gr1	PerCP-C5.5	RB6-8C5	BioLegend	108427
CD11c	PerCP-C5.5	N418	BioLegend	117327
CD11b	PerCP-C5.5	M1/70	BioLegend	101227
CD16/32	APC-CY7	93	BioLegend	101327
CD127	Pe-Cy7	A7R34	BioLegend	135013
CD34	BV421	SA376A4	BioLegend	152208
CD117	BV605	ACK2	BioLegend	135121
Sca-1	APC	E13-161.7	BioLegend	122511
CD150	PE	TC15-12F12.2	BioLegend	115904
Live/Dead	Zombie Aqua		BioLegend	423102

ChIP-qPCR

For ChIP-QPCR experiments approximately 6,000 GMPs from *C. scindens* colonized or control mice (CBA/J) were sorted on an Influx cell sorter into Cryostore CS10 (Stemcell) as above. The samples were thawed then subjected to chromatin isolation, chromatin shearing, DNA isolation,

and ChIP. ChIP-qPCR and data analysis was then preformed. *Chromatin Isolation:* Chromatin was isolated using the ChromaFlash Chromatin Extraction Kit (EpiGentek, Cat. #P-2001). *Chromatin Fragmentation:* Chromatin was sheared using the EpiSonic 2000 Sonication System (EpiGentek, Cat. #EQC-2000) based on the standard operation protocol for small amounts of cells. Chromatin was sonicated for 20 cycles with 45" On and 15" Off. *Chromatin Quantification:* Total volume of chromatin solution was 60 μl for each sample. The sheared chromatin concentration was measured by fluorescence quantification of chromatin associated DNA. *DNA Purification:* DNA was purified using 5 μl of sheared chromatin as input. DNA was eluted with 15 μl of water. 1 μl of purified DNA was used for fluorescence quantification. *Antibody validation:* The anti-H3K4me3 (Epigentek Cat. #A-4033), anti-H3K27me3 (Epigentek Cat. #A-4039) antibodies were validated using the Pre-Sure ChIP Antibody Validation Kit (EpiGentek, Cat. #P-2031). The ChIP-Grade Intensity (CGI) is 4.0 for H3K4me3, and 4.2 for H3K27me3, respectively. *Chromatin Immunoprecipitation:* Because of small amount of chromatin for each sample, the ChIP reaction was based on the high sensitivity ChIP protocol modified from P-2027 kit. 50 ng of sheared chromatin samples in 200 μl ChIP assay buffer were added into the wells coated with 0.5 μg of anti-H3K4me3 or anti-H3K27me3, respectively. 2 ug of Jurkat cell chromatin was used as a positive control. The samples were incubated at room temperature for 180 minutes with continuous shaking (100 rpm). After incubation, the wells were washed and the chromatin immunoprecipitated DNA was purified and eluted in 14 μl of water. *qPCR:* was performed in duplicate using 1μl of DNA and gene-specific primers designed for the target gene region for 60 cycles. Un-ChIPed DNA (10%) was used as input for determining enrichment efficiency (Input%). Primer sequences based on the targeted CEBPA

and CEBPB region sequences are listed below/ CEBPA-1: CEBPA-TSS; CEBPA-2: CEBPA-promoter; CEBPB-1: CEBPB-TSS; CEBPB-2: CEBPB-promoter

**Primer sequences Binding site**	**Forward**	**Reverse**
CEBPA-1	5’-TGCCGGGAGAACTCTAACT-3’	5'-TCTGGAGGTGACTGCTCAT-3'
CEBPA-2	5'-CGATCTCTCTCCACTAGCACT-3'	5'-CGCTTTTATAGAGGGTCGG-3'
CEBPB-1	5'-CCTTATAAACCTCCCGCTC-3'	5'-CTTCCATGGGTCTAAAGGC-3'
CEBPB-2	5'-GTAGCTGGAGGAACGATCTG-3'	5 '- T C G G GAACACGGAGGAG-3'

Colony forming assay for determination of bone marrow hematopoietic precursors

Bone marrow cells were isolated and then cultured in methylcellulose-based medium that included, 3 units/mL Epo, 10 ng/mL mouse recombinant IL-3, 10 ng/mL human recombinant IL-6, and 50 ng/mL mouse recombinant stem-cell factor per manufacturer procedures (M3434; StemCell Technologies, Vancouver, BC). Colony formation of burst-forming unit–erythroid (BFU-Es), colony-forming unit–granulocyte/monocyte (CFU-GMs), and CFU granulocyte/erythrocyte/monocyte/macrophage (CFU-GEMMs) were analyzed after 7 days. Experiments shown are representative of 2 experimental replicates.

RNA sequencing and data analysis

GMPs were isolated from six control and six *C. scindens*-treated mice as described above. RNA was isolated from approximately 7,000 sorted GMPs per mouse utilizing the Qiagen RNeasy Micro Kit. Ribosomal RNA depletion was performed using the NEBNext® rRNA Depletion Kit (Human/Mouse/Rat) and alternate protocol for low yield RNA. Directional cDNA libraries were generated the NEBNext® Ultra™ Directional RNA Library Prep Kit for Illumina®, including 15 PCR cycles of amplification. Multiplexed samples were sequenced (75 bp paired-end reads) using the NextSeq 500 platform. For sequencing, libraries were sequenced with the NGS NextSeq kit - 150 cycle High Output Kit, paired end 75x75 bp read. Data analysis was performed by the UVA Bioinformatics Core. Reads were mapped to the mouse transcriptome (GRCm38) using Salmon and gene level abundances quantified using tximport. Differential gene expression analysis was then performed in R using DESeq2[3], which yielded no differentially expressed genes with FDR-corrected p-values < 0.05. ConsensusPathDB [4] was used to identify functional enrichment in the top 50% of genes ranked by unadjusted P value. RNA-seq data are available from the Gene Expression Omnibus (accession number GSE121503).

16S rRNA Gene Amplicon Sequencing (Mouse)

DNA was isolated from mouse cecal lysate (QIAamp DNA Stool Mini Kit). The V4 region of the 16S rRNA gene was amplified from each sample using the dual indexing sequencing strategy as described previously[5]. Sequencing performed on the Illumina MiSeq platform, using a MiSeq Reagent Kit V2 500 cycles (Illumina cat# MS102-2003), according to the manufacturer's instructions with modifications found in the Schloss SOP: https://github.com/SchlossLab/MiSeq_WetLab_SOP. The mock community produced

ZymoBIOMICS Microbial Community DNA Standard (Zymo Research cat# D6306) was sequenced to monitor sequencing error. The overall error rate was 0.02% as determined using the software package mothur version 1.39.5 following the Illumina MiSeq standard operating procedure [6] . The sequences associated with analysis of the murine data were deposited to the SRA under the PRJNA503904.

16S rRNA Gene V4 region sequencing, sample selection and extraction (Human)

Diarrheal and non-diarrheal reference stools were collected during scheduled study visits (scheduled visits took place at enrollment and at 6, 10, 12, 14, 17, 18, 39, 40, 52, 53, 65, 78, 91, 104 weeks of age)[7]. Samples were brought into the study clinic and stool was transported from the field to our laboratory at 4°C, aliquoted in DNase- and RNase-free cryovials, and stored at -80°C on the day of collection. 200ug was removed for total nucleic acid extraction. Positive extraction controls were achieved by spiking phocine herpesvirus (Erasmus MC, Department of Virology, Rotterdam, The Netherlands) and bacteriophage MS2 (ATCC 15597B; American Type Culture Collection, Manassas, VA) into each sample during the extraction process. The fecal DNA was then tested for *E. histolytica* by use of a multiplex qPCR assay to detect parasitic protozoans as described by Haque et al (38). DNA Samples positive for *E. histolytica* and non-diarrheal reference samples were shipped to our laboratory at UVA for library construction.

Library construction for next-generation sequencing (Human)

The entire 255bp V4 region of the 16S rRNA gene was amplified as previously described[8], using phased Illumina-eubacteria primers to amplify the V4 region of 16S rRNA gene (515F – 806R) and to add the adaptors necessary for illumina sequencing and the GOLAY index necessary for de-multiplexing after parallel sequencing. Negative controls included the addition of extraction

blanks that were tested throughout the amplification and sequencing process to ensure they remained negative. As a positive PCR control, DNA extracted from the HM-782D Mock Bacteria Community (ATCC through BEI Resources) was run on each plate and added to the library. The library was then sent to UVA Biomolecular core facility. A PhiX DNA library was spiked into the 16S sequencing run (20%) to increase genetic diversity prior to parallel sequencing in both forward and reverse directions using the Miseq V3 kit and machine (per manufacturer's protocol).

16S rRNA Gene Amplicon Curation and Analysis (Human)

The 16S data curation and analysis from the human stool samples was performed using R version 3.5.1. Sequences were curated using the R package DADA2 version 1.10.1, following the DADA2 pipeline tutorial v1.8 [9] . Briefly, reads were filtered and trimmed using standard parameters outlined in the DADA2v1.8 pipeline. The error rates for the murine or human amplicon datasets were determined using the DADA2's implementation of a parametric error model. Samples were then dereplicated and sequence and variants were inferred. For the 16S data from murine samples, overlapping forward and reverse reads were merged and sequences that were shorter than 250bp or longer than 254bp were removed. For the 16S data from human samples, only forward reads were used. Finally, chimeras were removed. Taxonomy was assigned to amplicon sequence variants (ASVs) using the DADA2- formatted SILVA taxonomic training data release 132 [10]. A partial sequence from *[Lachnoclostridium] scindens ATCC 35704* (NCBI Reference Sequence: NR_028785.1) was added to the Silva training data v132 to attempt to identify *Clostridium scindens* ASVs. Following sequence curation, the packages phyloseq v1.26.1[11], vegan, dplyer and ggplot2 were used for analysis and generation of figures. This includes determining the axes for the PCoA plots of Bray-Curtis dissimilarities (beta-diversity) calculated from rarified sequence abundance. Additionally, the package vegan was used to

determine significant differences between groups with PERMANOVA. The sequences associated with analysis of the human data will be deposited to the SRA and linked via the dbGaP accession number phs001478.v1.p1**.** Full details of the design of the human cohort study have been described [7] and all studies were approved by the Ethical Review Committee of the ICDDR, B and the Institutional Review Boards of the Universities of Virginia and informed consent was obtained after the nature and possible consequences of the studies were explained in all cohort studies. Final figures were modified and arranged in Adobe Illustrator CC.

***C. scindens, A. muciniphila,* marrow and cecal lysate qPCR**

Purity of *C. scindens* culture was confirmed via qPCR and Sanger sequencing with broad specificity eubacteria primers, EUB forward 5'-ACTCCTACGGGAGGCAGCAGT-3'EUB reverse 5'-ATTACCGCGGCTGCTGGC-3', and *C. scindens* baiCD stereo-specific 7alpha/7beta-hydroxy-3-oxo-delta4-cholenoic acid oxidoreductase primers, BaiCD F - 5′-CAGCCCRCAGATGTTCTTTG-3′ BaiCD R - 5′-GCATGGAATTCHACTGCRTC-3′ *C. scindens* colonization was measured via qPCR from cecal lysate (QIAamp DNA Stool Mini Kit). qPCR for baiCD with SYBR green was performed and data were normalized to expression of a conserved *Eubacteria* 16s RNA gene (EUB) [12]. Primer concentrations, annealing temperatures, and cycle number were optimized for each primer pair. For each primer pair, a dilution curve of a positive cDNA sample was included to enable calculation of the efficiency of the amplification. For *A. muciniphila* the probe: /5HEX/ ACAGAGGTCTCAAGCGTTGTTCGG /3BHQ_2/ and primer pairs , Forward: AGAGGAAGAGACGGCTAACT Reverse: ACGACTTACGAAACAGCCTAC were utilized along with Bio-Rad iQ Multiplex Powermix (Cat. No. 172-5848) and a TM of 60˚C. The relative message levels of each target gene were then normalized to EUB or the mouse housekeeping

gene S14 using a method described and utilized previously [13–15]. Data is presented as relative expression. For sorted marrow qPCR, RNA was isolated from approximately 7,000 sorted GMPs utilizing the Qiagen RNeasy Micro Kit. S14 forward 5’-TGGTGTCTGCCACATCTTTGCATC-3’, S14 reverse 5’ AGTCACTCGGCAGATGGTTTCCTT-3’,Jmjd3 forward 5’-CTCTGGAACTTTCATGCCGG-3’ Jmjd3 reverse, 5’-CTTAGCCCCATAGTTCCGTTTG-3,’ CebpA forward 5’-CAAAGCCAAGAAGTCGGTGGACAA, CebpA reverse 5’ -TCATTGTGACTGGTCAACTCCAGC

CebpE forward 5’-TGTGGGCACCAGACCCTAAG, CebpE reverse 5’-GCTGCCATTGTCCACGATCT, CebpD forward 5’- CTTTTAGGTGGTTGCCGAAG, CebpD reverse 5’ GCAACGAGGAATCAAGTTTCA, Kdm4dl forward 5’-CATGGTCACCTTTCCCTATGG, Kdm4dl reverse 5’-AAAATTGATGGCCTCTGCG. Primers were purchased from Integrated DNA Technologies Coralville, Iowa, USA.

Serum deoxycholate ELISA in Children and Mice

Serum Deoxycholate was measured via ELISA (Cloud-Clone Corp. CES089Ge) in 80 children each from two birth cohorts in Mirpur Dhaka, Bangladesh. Full details of the design of these two birth cohort studies, including socioeconomic status data, have been described [7,16] and all studies were approved by the Ethical Review Committee of the ICDDR, B and the Institutional Review Boards of the Universities of Virginia and informed consent was obtained after the nature and possible consequences of the studies were explained in all cohort studies. Children were approximately two years of age and serum was selected by identifying diarrheal stools within 6 months of the blood draw that were *E. histolytica* positive (n=40) and negative (n=40) as

identified via qPCR in both cohorts [17]. Association of serum DCA with intestinal *E. histolytica* infection was evaluated in a logistic regression, adjusting for HAZ at enrollment, monthly family expenditure, and mother's education. Serum was measured in 6 mice from each of at least two experiments that were gavaged with *C. scindens* or media control as described utilizing the same kit or in DCA treated mice. For both children and mice 25uL of serum was utilized at a 1:2 (humans) or 1:4 (mice) dilution following kit protocol with a 10 minute development step following administration of substrate solution. The logistic regression analyses were performed using the function "glm" in R software version 3.5.2 (r-project.org), while model fitting and predictability were evaluated using the R packages "lmtest" and "pROC" respectively.

Serum ALT in mice

Serum ALT was measured via ELISA (CloudClone SEA207Mu). Serum from DCA and PBS treated mice were assayed with the ELISA at a dilution of 1:3 in PBS. The protocol provided in the ELISA kit was utilized and unaltered. Linear regression was done in Microsoft Excel with an r^2 value >0.99. Data analysis was performed in Graphpad Prism.

Cecal Lipocalin in mice

Cecal Lipocalin was measured via ELISA (R&D DY1857-05). Ceca from DCA and PBS treated mice were homogenized in 1 mL of PBS in Lysing Matrix F (MP Biomedicals 116915050-CF) for 1 minute. The supernatant was then assayed at neat, 1:2, and 1:10 dilutions. The protocol provided in the ELISA kit was utilized and unaltered. Linear regression was done in Microsoft Excel with an r^2 value >0.99. Data analysis was performed in Graphpad Prism.

Targeted profiling of serum bile acids using UPLC-MS

In specific pathogen free mice, Plasma bile acids were quantified as previously described [18] by ACQUITY ultraperformance liquid chromatography (UPLC) (Waters, Ltd., Elstree, UK). Briefly, 100 μl of plasma were spiked with isotopically labeled bile acid standards followed by the addition of 300 μl of ice-cold methanol to facilitate protein precipitation. Bile acids were separated over a 15-minute gradient on an ACQUITY BEH C8 column (1.7 μm, 100 mm x 2.1 mm), detected by a Xevo TQ-S mass spectrometer (Waters, Manchester, UK) operating in the negative ionization mode (ESI-) and assayed using multiple reaction monitoring (MRM).

Supplemental References

1. Houpt ER, Glembocki DJ, Obrig TG, et al. The mouse model of amebic colitis reveals mouse strain susceptibility to infection and exacerbation of disease by CD4+ T cells. *J Immunol*. 2002;169(8):4496-4503. http://www.ncbi.nlm.nih.gov/pubmed/12370386. Accessed May 29, 2013.
2. Swamydas M, Lionakis MS. Isolation, purification and labeling of mouse bone marrow neutrophils for functional studies and adoptive transfer experiments. *J Vis Exp*. 2013;(77):e50586. doi:10.3791/50586
3. Love MI, Huber W, Anders S. Moderated estimation of fold change and dispersion for RNA-seq data with DESeq2. *Genome Biol*. 2014;15(12):550. doi:10.1186/s13059-014-0550-8
4. Herwig R, Hardt C, Lienhard M, Kamburov A. Analyzing and interpreting genome data at the network level with ConsensusPathDB. *Nat Protoc*. 2016;11(10):1889-1907. doi:10.1038/nprot.2016.117
5. Kozich JJ, Westcott SL, Baxter NT, Highlander SK, Schloss PD. Development of a dual-index sequencing strategy and curation pipeline for analyzing amplicon sequence data on the MiSeq Illumina sequencing platform. *Appl Environ Microbiol*. 2013;79(17):5112-5120. doi:10.1128/AEM.01043-13
6. Schloss PD, Westcott SL, Ryabin T, et al. Introducing mothur: open-source, platform-independent, community-supported software for describing and comparing microbial communities. *Appl Environ Microbiol*. 2009;75(23):7537-7541. doi:10.1128/AEM.01541-09
7. Kirkpatrick BD, Colgate ER, Mychaleckyj JC, et al. The “Performance of Rotavirus and Oral Polio Vaccines in Developing Countries” (PROVIDE) Study: Description of Methods of an Interventional Study Designed to Explore Complex Biologic Problems. *Am J Trop Med Hyg*. 2015;92(4):744-751. doi:10.4269/ajtmh.14-0518
8. Caporaso JG, Lauber CL, Walters WA, et al. Ultra-high-throughput microbial community analysis on the Illumina HiSeq and MiSeq platforms. *ISME J*. 2012;6(8):1621-1624. doi:10.1038/ismej.2012.8
9. Callahan BJ, McMurdie PJ, Rosen MJ, Han AW, Johnson AJA, Holmes SP. DADA2: High-resolution sample inference from Illumina amplicon data. *Nat Methods*. 2016;13(7):581-583. doi:10.1038/nmeth.3869
10. Pruesse E, Quast C, Knittel K, et al. SILVA: a comprehensive online resource for quality checked and aligned ribosomal RNA sequence data compatible with ARB. *Nucleic Acids Res*. 2007;35(21):7188-7196. doi:10.1093/nar/gkm864
11. McMurdie PJ, Holmes S. phyloseq: An R Package for Reproducible Interactive Analysis and Graphics of Microbiome Census Data. Watson M, ed. *PLoS One*. 2013;8(4):e61217. doi:10.1371/journal.pone.0061217
12. Hoffman PS, Marshall AM, Olekhnovich I, et al. Preclinical Studies of Amixicile: A Systemic Therapeutic Developed for Treatment of Clostridium difficile Infections also shows Efficacy against Helicobacter pylori. *Antimicrob Agents Chemother*. June 2014. doi:10.1128/AAC.03112-14
13. Muller PY, Janovjak H, Miserez AR, Dobbie Z. Processing of gene expression data generated by quantitative real-time RT-PCR. *Biotechniques*. 2002;32(6):1372-1374, 1376, 1378-1379. http://www.ncbi.nlm.nih.gov/pubmed/12074169. Accessed June 17, 2016.
14. Burgess SL, Buonomo E, Carey M, et al. Bone Marrow Dendritic Cells from Mice with an

Altered Microbiota Provide Interleukin 17A-Dependent Protection against Entamoeba histolytica Colitis. *MBio*. 2014;5(6):e01817-14-. doi:10.1128/mBio.01817-14

15. Burgess SL, Saleh M, Cowardin CA, et al. Role of Serum Amyloid A, GM-CSF and Bone Marrow Granulocyte-Monocyte Precursor Expansion in Segmented Filamentous Bacteria-mediated Protection from *Entamoeba histolytica* . *Infect Immun*. July 2016:IAI.00316-16. doi:10.1128/IAI.00316-16
16. Mondal D, Minak J, Alam M, et al. Contribution of enteric infection, altered intestinal barrier function, and maternal malnutrition to infant malnutrition in Bangladesh. *Clin Infect Dis*. 2012;54(2):185-192. doi:10.1093/cid/cir807
17. Roy S, Kabir M, Mondal D, Ali IKM, Petri WA, Haque R. Real-time-PCR assay for diagnosis of Entamoeba histolytica infection. *J Clin Microbiol*. 2005;43(5):2168-2172. doi:10.1128/JCM.43.5.2168-2172.2005
18. Sarafian MH, Lewis MR, Pechlivanis A, et al. Bile Acid Profiling and Quantification in Biofluids Using Ultra-Performance Liquid Chromatography Tandem Mass Spectrometry. *Anal Chem*. 2015;87(19):9662-9670. doi:10.1021/acs.analchem.5b01556
